# Point-of-care-ready nanoscale ISFET arrays for sub-picomolar detection of cytokines in cell cultures

**DOI:** 10.1007/s00216-020-02820-4

**Published:** 2020-07-28

**Authors:** Dipti Rani, Yogesh Singh, Madhuri Salker, Xuan Thang Vu, Sven Ingebrandt, Vivek Pachauri

**Affiliations:** 1 Department of Computer Sciences and Microsystem Technology, University of Applied Sciences Kaiserslautern, Amerikastrasse 1, 66482 Zweibruecken, Germany; 2 Institute of Medical Genetics and Applied Genomics, Eberhard-Karls University Tuebingen, Calwerstraße 7, 72076 Tübingen, Germany; 3 Women’s Hospital, Eberhard-Karls University Tuebingen, Calwerstraße 7/6, 72076 Tübingen, Germany; 4 Institute of Materials in Electrical Engineering 1 (IWE1), RWTH Aachen University, Sommerfeldstrasse 24, 52074 Aachen, Germany

**Keywords:** Ion-sensitive field-effect transistors, Silicon nanowires, Immunosensor, Cytokines, Label-free

## Abstract

Rapid and frequent screening of cytokines as immunomodulation agents is necessary for precise interventions in severe pathophysiological conditions. In addition to high-sensitivity detection of such analytes in complex biological fluids such as blood, saliva, and cell culture medium samples, it is also crucial to work out miniaturized bioanalytical platforms with potential for high-density integration enabling screening of multiple analytes. In this work, we show a compact, point-of-care-ready bioanalytical platform for screening of cytokines such as interleukin-4 (IL-4) and interleukin-2 (IL-2) based on one-dimensional ion-sensitive field-effect transistors arrays (nanoISFETs) of silicon fabricated at wafer-scale via nanoimprint lithography. The nanoISFETs biofunctionalized with receptor proteins alpha IL-4 and alpha IL-2 were deployed for screening cytokine secretion in mouse T helper cell differentiation culture media, respectively. Our nanoISFETs showed robust sensor signals for specific molecular binding and can be readily deployed for real-time screening of cytokines. Quantitative analyses of the nanoISFET-based bioanalytical platform was carried out for IL-4 concentrations ranging from 25 fg/mL (1.92 fM) to 2.5 μg/mL (192 nM), showing a limit of detection down to 3–5 fM, which was found to be in agreement with ELISA results in determining IL-4 concentrations directly in complex cell culture media.

**Electronic supplementary material:**

The online version of this article (10.1007/s00216-020-02820-4) contains supplementary material, which is available to authorized users.

**Figure:**
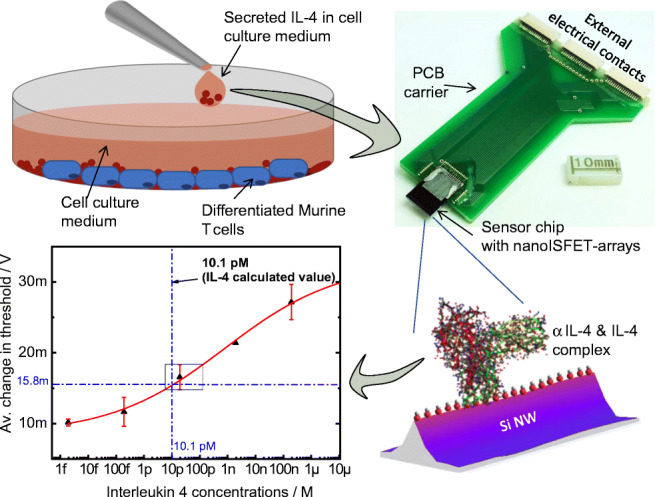
Graphical abstract

## Introduction

Cytokines are secreted by immune cells such as lymphocytes (T cells) and epithelial cells, as low molecular weight proteins which are responsible for management of various host defense mechanisms. In response to an infection, specialized T cells change their function and provide biomolecular triggers to the immune system by initiating a pro-inflammatory cascade, i.e., excessive production of pro-inflammatory cytokines including interleukin-2 (IL-2), interleukin-6 (IL-6), interferon gamma (IFNγ), and tumor necrosis factor alpha (TNF alpha). In a subsequent anti-inflammatory phase, the immune response switches to production of anti-inflammatory cytokines, including interleukin-10 (IL-10), transforming growth factor (TGF-β), and interleukin-4 (IL-4) [1]. Secretion profiles of different cytokines are therefore important biomarkers in disease management and immunology studies [2].

The cytokine IL-4 regulates differentiation, proliferation, and apoptosis hematopoietic and non-hematopoietic cells [3, 4]. The biosignalling machinery of IL-4 is known to be very diverse. For instance, IL-4 plays a crucial role in differentiation of naïve CD4+ T cells (or Th cells) by promoting Th2 cell differentiation acting against parasitic infections and rheumatoid arthritis [1, 2]. IL-4 is, conversely, involved in promoting diseases such as allergic asthma [2]. Similarly, IL-2 have essential role in key immune response mechanisms where they directly impact the differentiation of T cells, effector T cells, and memory T cells, helping the immune system act against infection [5, 6]. Expression of IL-2 is tightly regulated for their role in maintaining transient feedback loops controlling the immune response and also playing a key role in cell-mediated immunity [4, 6]. Due to highly dynamic and heterogeneous secretion profiles in different tissues, precise monitoring of cytokine during a disease progression and treatment pose a real challenge. Generally, labelling-based methods are performed for qualitative tissue analysis while enzyme-linked immunosorbent spot (ELISpot) and enzyme-linked immunosorbent assay (ELISA) are employed for quantitative analysis [1, 7]. Though high sensitivity and multiplexing ability, ELIspot and ELISA are not suitable for rapid immune status monitoring and generally require several days before completion [1, 7, 8]. Given ultrafast and efficient screening of cell-secreted cytokines, personalized care in different inflammatory conditions, such as sepsis, cancer, lupus, and graft-versus-host disease (GVHD), is expected to be critically improved [7, 9].

Point-of-care (PoC) immunodiagnostic platforms based on miniaturized optical and electrical biosensors and smart instrumentation such as real-time readout, data transfer, and analysis tools are in the rise [10, 11]. Such smart PoCs, when integrated in high-density arrays, have the potential to revolutionize the immune profiling technology in the laboratory and clinical environments. In recent years, mechanical (quartz crystal microbalance), optical (fluorescence, colorimetric, photonic resonators, and Raman etc.), and electrical (electrochemical, impedance and field-effect based) sensor platforms have been realized for label-free screening of cytokines [7–9, 12–22]. Notably, localized surface plasmon resonance (LSPR)-based multi-arrayed biosensors were shown for label-free, real-time detection of IL-2, IL-4, IL-6, IL-10, TNF-α, and IFNγ from serum [9, 23]. Most of the optical methods, however, still require expensive instrumentation and are not suitable for PoC integration [7, 8, 24]. Among electrical biosensors, one-dimensional (1D) and two-dimensional (2D) ion-sensitive field-effect transistors (ISFETs) based on silicon (Si), graphene, and molybdenum disulfide (MoS_2_) have been employed for detection of cytokines such as TNF-α, IL-1β, IL-6, and IL-8 [12, 25–35]. A comparative analysis (in terms of sensitivity and response time) of different electrical sensors realized for detection of cytokines in last 5 years is listed in Table 1. Here, top-down fabricated electrical biosensors such as Si-based ISFETs show a real promise for high-throughout readout for detection of ultralow concentrations of cytokines due to the highly identical sensor behavior and due to their reliable multiplexed detection capabilities [46, 47].

**Table 1:** Comparison of label-free electrical biosensors for screening of cytokines within the last 5 years

	Analyte	Approach	LoD	Range	Response time	Remarks	Ref.
1	IFNγ, IL-10	Impedance spectroscopy	25 and 46 pg/mL	0.1–5, and 0.1–2 ng/mL	30 min, incubation time	Aerosol-jet printed graphene-based films modified with IFNγ, IL-10 antibodies	[36]
2	TNF-α	Impedance spectroscopy	0.085 and 2 pg/mL	-	90 min, incubation time	Screen printed gold electrodes with anti-TNF-α for detection in tears and blood serum	[37]
3	IL-6	Impedance spectroscopy	0.33 pg/mL	1–15 pg/mL	30 min, incubation time	Gold and polypyrrole NP-based aptasensor	[38]
4	IFNγ, TNF-α	Electrochemical	-	From 300 to 50,000 T cells	-	Nanowire aptasensors for detection of cell-secreted cytokines	[32]
5	TNF-α	Electrochemical	100 pg/mL	100 pg/mL −100 ng/mL	20 min, incubation time	Polycarbonate-modified gold microelectrodes, detection in whole serum	[39]
6	VEGF	Electrochemical	1 pg/mL	10–500 pg/mL	60 min, incubation time	GO-modified glassy carbon electrodes	[40]
7	IL-6	Electrochemical	10 pM		1 h, incubation time	IL-6-specific receptors functionalized on PEDOT:PSS devices, in physiological buffers	[41]
8	IL-8	Electrochemical	90 fg/mL	900 fg/mL–900 ng/mL	15 min incubation time	Polyethyleneglycolated gold electrodes functionalized with IL-6 receptors	[20]
9	IL-1, IL-10	Electrochemical, Impedance	0.3, 0.7 pg/mL	1–15 pg/mL	30 min, incubation time	Fully integrated microelectrode arrays	[13]
10	TNF-α	Chronoamperometry	1 pM	1–30 pM	30 min, incubation time	TNF-α-specific antibodies on gold, detection in human saliva	[42]
11	IL-6	FET	1.37 pg/mL	-	15 min, incubation time	IL-6-specific receptor antibodies on CNT devices	[25]
12	TNF-α	FET	1 pg/mL	100 fg/L to 100 pg/L	100 s, Real-time	CNT and floating metal electrodes modified with anti-TNF-α	[34]
13	IL-2	FET	10–14 fM	20 fM–200 pM	3.5 h, incubation time	Si NW-based devices for indirect sandwich immunoassay combined with DNA amplification	[31]
14	TNF-α	FET	1 pM	1 pM–10 nM	-	Electrolyte gated organic field-effect transistors (EGOFETs)	[43]
15	TNF-α	FET	5 pM		-	Graphene on flexible ultrathin Mylar substrates for wearable sensors	[44]
16	TNF-α	FET	26 pM	0.05–50 nM	5 min, real-time	TNF-α-specific aptamer as biofunctional layer on graphene-based flexible devices	[45]
17	TNF-α	FET	60 fM	6 fM–6 pM	2 h, incubation time	TNF-α-specific receptor antibodies on few-layer MoS_2_ devices	[35]
18	IL-4, IL-2	FET	3–5 fM	3 fM–1 μM	15 min, real-time	nanoISFETs modified with IL-4/IL-2-specific antibodies, in T cell culture, also real-time detection	This work

In this work, top-down fabricated nanoscale ISFET-arrays (nanoISFETs) of Si are shown as a proof-of-concept PoC-ready biosensor platform for label-free electrical detection of cell-secreted IL-4 and IL-2. Fluidics encapsulated nanoISFETs were surface modified with IL-4- and IL-2-specific receptor molecules and deployed for field-effect-based detection. The field-effect behavior of the sensor platform was thoroughly characterized upon biofunctionalization and analyte binding steps. The biosensor platform was also successfully deployed for real-time screening of cytokines. The results obtained through electrical screening of IL-4 were found to be in agreement with those obtained from standard ELISA as a reference method. Therefore, nanoISFET array-based sensor platforms such as presented here are apt for further deployment as integrated total bioanalytical systems for screening of immune balance in PoC approaches.

## Materials and methods

### IL-4 and IL-2

The cytokines IL-4 and IL-2 used in this work were secreted from T helper cells into their culture medium. Isolation of naïve T cell from the spleen and lymph nodes of mice and their differentiation was based on our earlier works [48, 49]. The method used for isolation and differentiation of Th cells for expression of IL-4 and IL-2, and for minimal expression of cytokines for control experiments, is discussed in the Electronic Supplementary Material (ESM, section S1). Anti-IL-4 and anti-IL-2 antibodies used as receptors towards specific binding of IL-4/IL-2 expressed in the cell culture media were obtained from eBiosciences (Thermo Fisher), Germany. A recombinant IL-4 protein specific to anti-IL-4 was also commercially procured (from eBioscience, Germany) in order to ascertain the molecular binding and sensor performance. In addition, recombinant IL-2 protein (eBioscience, Germany) non-specific to the anti-IL-4 was obtained for control experiments. Ten millimolar phosphate buffer saline (PBS) with pH 7.4 was used for all the samples with commercially procured biomolecules.

### Assembly of the biosensor platform

**Fig. 1:**
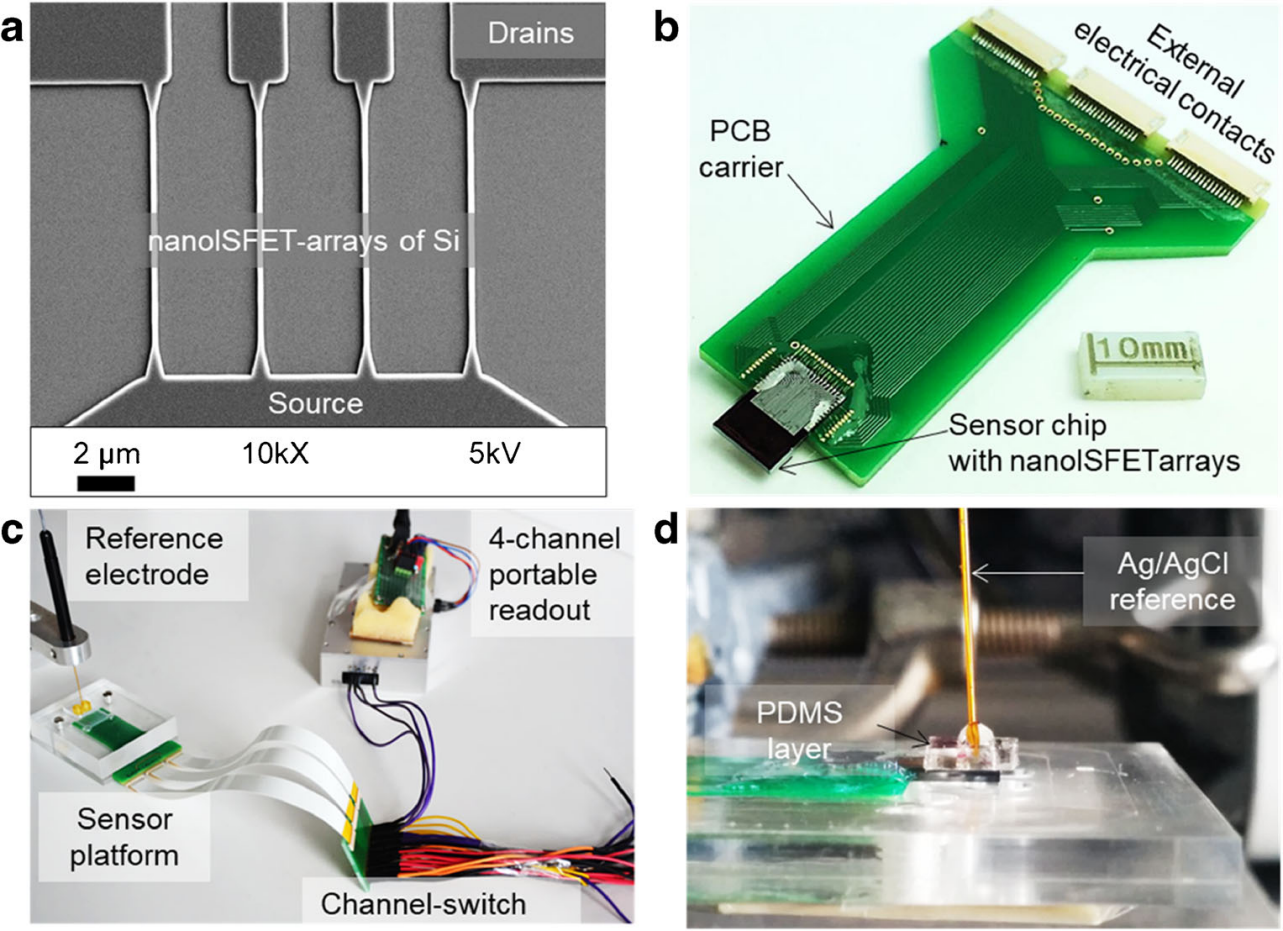
Nanoscale silicon ISFETs for the assembly of a point-of-care electrical biosensor for monitoring of cytokines. (**a**) Scanning electron micrograph image of one set of 4 nanoISFETs with common source and individual drain electrodes; (**b**) photograph of a wire-bonded and encapsulated sensor chip ready for biosensor measurements. (**c**, **d**) Photographs showing a sensor chip connected to the miniaturized DC readout system

NanoISFET arrays of silicon used for the assembly of the electrical biosensor platform were fabricated in a top-down lithography process. A combination of nanoimprint lithography, wet-chemical etching, and photolithography was used to cast out Si nanowires (NWs) in the top Si layer of prime quality silicon-on-insulator (SOI) wafers (procured from SOITec, France). The fabrication protocol for realization of nanoISFETs with near-identical biosensor characteristics was reported earlier [50]. Such nanoISFET arrays were earlier deployed as an aptamer-based sensor platform for label-free detection of prostate-specific antigens (PSA)—a biomarker for the onset of prostate cancer [51]. Assembly of nanoISFET array-based miniaturized sensor platform used in this work is illustrated in Fig. 1. Individual NWs in the arrays form a trapezoidal cross section due to anisotropic etching and measure 14 μm in length, 250 nm in width, and 60 nm in height. Each sensor chip consists of 8 sets of such NWs (a total of 32 NWs) connected to a common source but individually addressable drain electrodes. Eight-millimeter silicon dioxide (SiO_2_) layer serves as an dielectric on top of the NWs as well as a passivation layer in the contact line regions. As shown in Fig. 1b, sensor chips were mounted on a chip carrier and wire-bonded. Polymethylmethacrylate (PMMA)- and polydimethoxysiloxane (PDMS)-based fluidic layers were used in order to facilitate efficient handling of small sample volumes (20 μl) and to protect the electrical contacts from the liquid environment. Figure 1c shows the complete measurement setup, connected to a miniaturized readout system via a channel switch. A portable four-channel device built around a 32-bit PIC microcontroller as the readout tool [52]. The readout is equipped with a user-friendly LabView-based software interface, communicating with the hardware to record and display the electrical measurements on a computer screen. In Fig. 1d, the sensor platform is shown from the side (without the top PMMA cover) with a miniaturized, electrochemical silver/silver chloride (Ag/AgCl) reference electrode (World Precision Instruments Europe, DRI-Ref, 450 μm diameter), which was used to apply the gate voltage (Vg) to the electrolyte solution.

### Biofunctionalization of interleukin-specific receptors

**Fig. 2:**
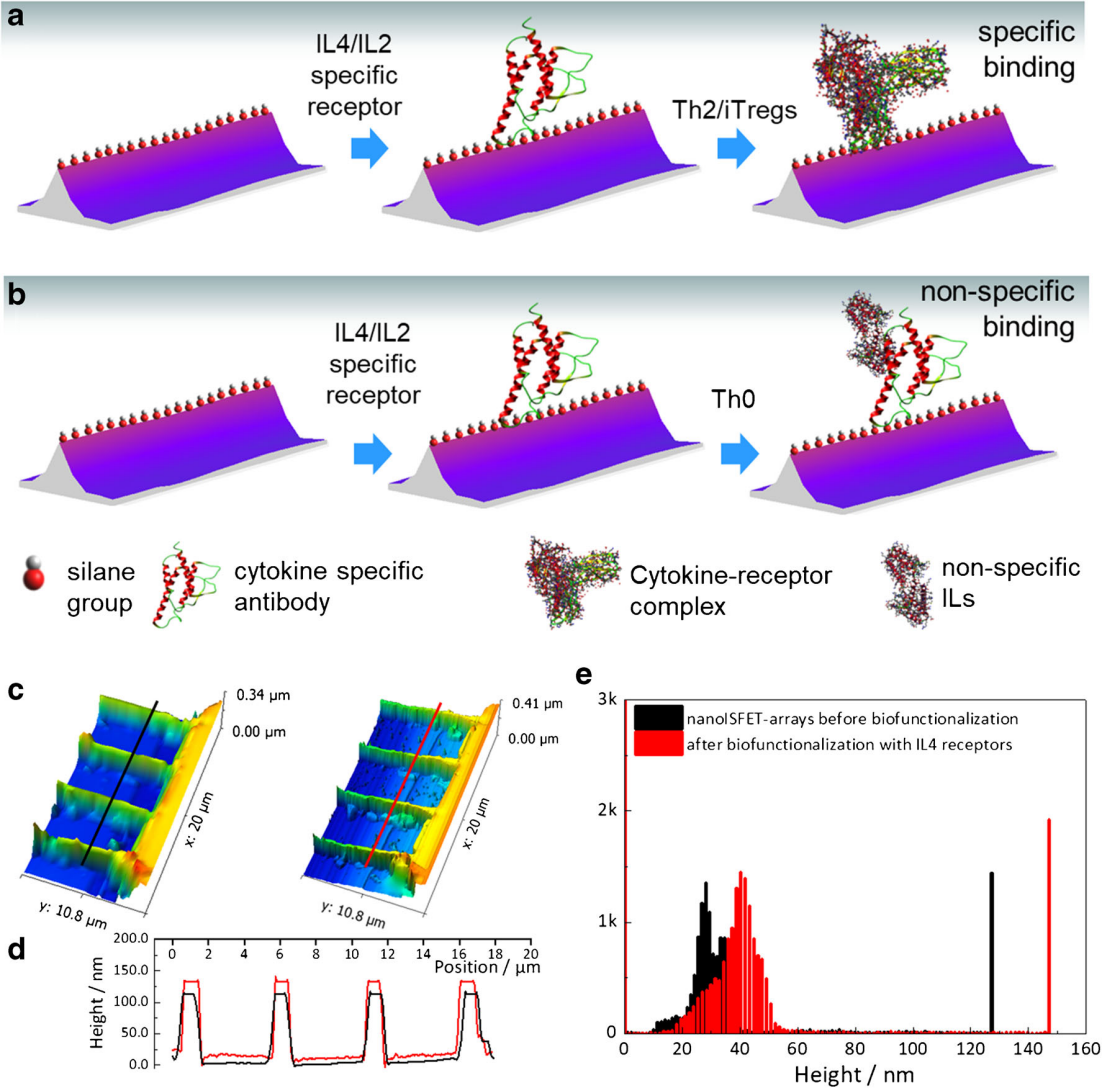
Biofunctional interface development for the label-free detection of interleukins using nanoISFETs. (**a**) IL-4 or IL-2-specific receptor molecules (anti-IL-4 or alpha IL-4 antibody and anti-IL-2 respectively) functionalized on the nanowire surface providing biospecific interaction for IL-4 and IL-2 from mouse T cell culture, (**b**) Th0 cultures with negligible concentrations of IL-4 and IL-2 were used for control experiments, (**c**) 3D illustrations of AFM measurements showing 4 nanowires before and after biofunctionalization, (**d**) graphs showing changes in the height profiles of nanoISFETs, and (**e**) histogram showing roughness analysis representing overall differences in heights for the scanned area shown, signifying the binding of receptor anti-IL-4

The strategy to render the nanoISFETs biologically specific towards binding of IL-4 and IL-2 is illustrated in Fig. 2a and b. NWs were functionalized with 1 μg/mL of anti-IL-4 and anti-IL-2 antibodies (obtained from eBioscience, Germany) in a surface modification process similar to other biofunctionalization strategies reported in previous works [50, 51, 53]. Briefly, gas-phase silanization with (3-glycidyloxypropyl)triethoxysilane (GPTES—Sigma-Aldrich, Germany) was used for surface modification of the nanoISFET surfaces and the immobilization of the IL-specific antibodies was done overnight at 37 °C. Control experiments for non-specific interactions were carried out using Th0 cultures with minimal expressions of IL-4 and IL-2. Further, control experiments evaluating non-specific adsorption of IL-4 on nanoISFET surface were also performed, which are included in the ESM (Fig. S3).

## Results and discussions

The biofunctionalization of nanoISFETs with cytokine-specific receptor molecules was confirmed by atomic force microscopy (AFM) as shown in Fig. 2c. The three-dimensional AFM scan images in the left and right compare the topography of ISFETs before and after the anti-IL-4 biofunctionalization steps. The graph in Fig. 2d compares height profiles of 4 nanoISFETs. A height increase of about 15 nm was measured after the biofunctionalization step. Further, Fig. 2e compares roughness of the measured areas before and after biofunctionalization in the histogram. This histogram counts the number of events recorded for the heights in the scan areas and up to 160 nm. For the nanoISFETs before functionalization, maximum numbers of events are recorded for heights around 26 nm and for a height of 130 nm. After the biofunctionalization, this event distribution shifts to around 40 nm and thereafter for a height of 146 nm. Increase in the number of events from 30 to 40 represents deposition of biomolecules in the chip surface, while from 130 to 146 represents an increase in the height of NWs.

**Fig. 3:**
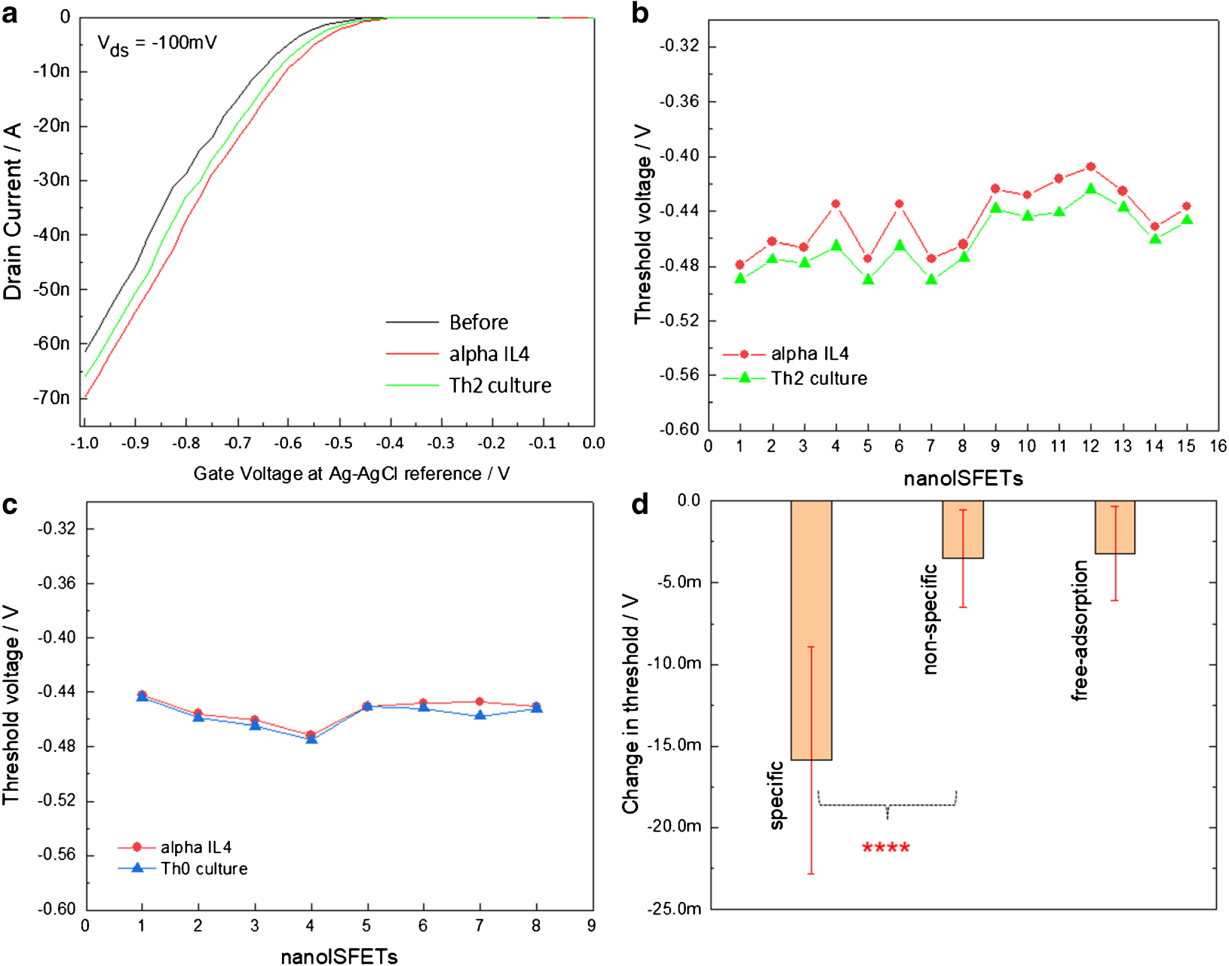
Electrical detection of IL-4 in complex mouse T cell culture medium. (**a**) Graph showing the transfer characteristics of an exemplary nanoISFET before and after biofunctionalization with anti-IL-4 (black and red curves), and after exposure to the culture medium with IL-4, (**b**) graph plots of Vth values associated with anti-IL-4-modified nanoISFETs (red) and after specific binding of IL-4 (green), (**c**) graph comparing threshold values for anti-IL-4-modified nanoISFETs (red) and mouse T cell culture media with negligible presence of IL-4 (blue), and (**d**) the graph showing average changes in the Vth values of anti-IL-4-modified nanoISFETs for specific binding (culture with IL-4) and non-specific binding (culture with other cytokines but negligible concentrations of IL-4) and free adsorption of IL-4s on non-modified nanoISFETs. An unpaired *T* test analysis of mean values for specific and non-specific binding showed the sensor signal as statistically extremely significant with *P* values of 0.0001

Sensor measurements were carried out by measuring the changes in threshold voltages (Vth) of the nanoISFETs after the biofunctionalization and upon placing small volumes of culture media with IL-4 and IL-2. Figure 3 sums up the sensor measurements carried out for the detection of IL-4 from the culture media. Figure 3a shows typical field-effect or transfer characteristics of a nanoISFET at the three stages, i.e., after surface modification with GPTES (or also mentioned as “before,” where the field-effect curve is shown in black), after the biofunctionalization (red curve), and in the cell culture medium with IL-4 cytokines (green curve). In the measurement graphs, the drain current (Id) is plotted against the gate-source voltage (Vgs) (0 to − 2 V) while applying a drain source bias (Vds) of − 100 mV. Successive field-effect measurements followed the same parameters unless specifically mentioned. Vth values for the field-effect curves were calculated from the first-order derivatives by extrapolating the linear regions of the curves. When comparing the linear regions of the field-effect curves, a shift towards a more positive Vg can be noticed after anti-IL-4 biofunctionalization. This shift towards more positive Vg is expected for anti-IL-4, because it represents the alpha chain of the IL-4 protein receptors, which is a 25-kDa transmembrane protein with an isoelectric point (pI) around 6 adding negatively charged functional groups onto the nanoISFET surfaces. Upon exposure to the culture medium with IL-4, the field-effect curves shift towards negative gate voltages. This shift is related to the specific binding of IL-4 (a 17.5-kDa protein) with pI value of around 9.1 bringing an overall increase of net positive charges at the interface [58]. Figure 3b shows the threshold values of 15 nanoISFETs associated with this molecular recognition event at the NW surface. The Vth values generally increase upon exposure to the culture media (green curve with symbols) with IL-4 as a result of IL-4 recognition at the nanoISFET surface. Further, in order to confirm molecular recognition at the nanoISFET surface, measurements were carried out in Th0 culture medium (with negligible concentrations of IL-4 among other ILs). Results from such measurements are shown in Fig. 3c, where threshold values are shown with the blue symbol. These curves represent the non-specific binding effect of ILs, which was remarkably lower than that for the specific binding. An overall comparison between the specific and non-specific binding is shown in the graph of Fig. 3d. For specific binding, an average change in threshold voltage was calculated to − 15.8 (± 6.9) mV against − 3.5 (± 3) mV for the non-specific binding. A statistical comparison between specific and non-specific binding using unpaired *T* test analysis suggested extremely significant signal with *P* value equivalent to 0.0001. In addition, we also carried out control measurements characterizing the free adsorption, where changes in Vth values were recorded for completely non-modified nanoISFETs in Th2 culture medium. While average changes in the Vth values − 3.2 (± 2) mV are shown in Fig. 3d, more detailed measurements are included in the ESM. With these measurements, we confirmed that anti-IL-4 antibody bioimmobilized nanoISFETs act as robust electrical biosensor platform, distinguishing specific molecular interactions even directly in the complex culture media. Even though it is difficult to access the exact molecular conformations and distributions of charges in IL-4 complexes (anti-IL-4 antibody-IL-4-antigen) with respect to their position within or outside the Debye length, regular changes in Vth values suggest for significant changes in surface charge density of biofunctional layer resulting in reproducible sensor signals.

**Fig. 4:**
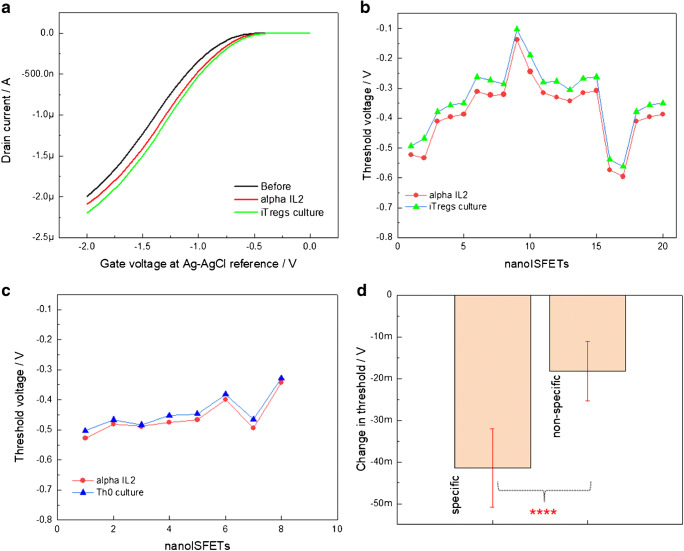
Electrical detection of IL-2 in complex mouse T cell culture medium (iTregs; differentiated using 2.5 ng/mL TGF-b and 5 ng/mL IL-2). (**a**) Graph showing typical transfer characteristics of an exemplary nanoISFET before and after biofunctionalization with anti-IL-4 (black and red curves), and after exposure to the culture medium with IL-2, (**b**) graph plots of Vths associated with alpha IL-2-modified nanoISFETs (red) and after specific binding of IL-2 (green), (**c**) graph comparing threshold values for anti-IL-2-modified nanoISFETs and mouse T cell culture with negligible presence of IL-2, and (**d**) the graph showing average changes in Vth of anti-IL-2-modified nanoISFETs for specific binding (culture with IL-2) and non-specific binding (culture with other cytokines, but negligible concentrations of IL-2). An unpaired *T* test analysis of mean values for specific and non-specific binding showed the sensor signal as statistically extremely significant with *P* values of 0.0001

Deploying nanoISFETs as an immunosensor platform as established from label-free detection of IL-4s in culture medium, we further modified the nanoISFETs with anti-IL-2 receptors. Representative field-effect measurements from a nanoISFET are shown in Fig. 4a. Field-effect curves in black, red, and green curves represent the successive steps of surface modification, biofunctionalization, and exposure to IL-2 containing culture medium, respectively (iTregs—which was differentiated using 2.5 ng/mL TGF-β and 5 ng/mL IL-2) [54]. With the biofunctionalization, the field-effect curve shifts towards positive gate voltages. After the exposure to culture medium with IL-2, the curve further shifts towards positive gate voltages. The Vth values associated with the shift of field-effect curves representing specific binding between alpha IL-2 and IL-2 are shown in Fig. 4b. A total of 20 nanoISFETs were measured which showed a Vth shift towards positive gate voltages. In addition, control measurements carried out with Th0 culture medium with negligible concentration of IL-2 did not show significant changes in Vth (Fig. 4c). A comparison for specific binding and control experiment is made by plotting the average changes in Vth values (Fig. 4d) for 8 nanoISFETs. For IL-2 detection, the average change in Vth was at − 41.4 (± 9) mV, while it was only − 18.2 (± 7) mV for the control experiments. Statistical comparison between specific and non-specific binding using unpaired *T* test analysis suggested extremely significant signal with *P* value equivalent to 0.0001. Normalized threshold voltage changes as sensor response upon specific and non-specific binding of IL-4 and IL-2 are included in ESM (Fig. S4).

**Fig. 5:**
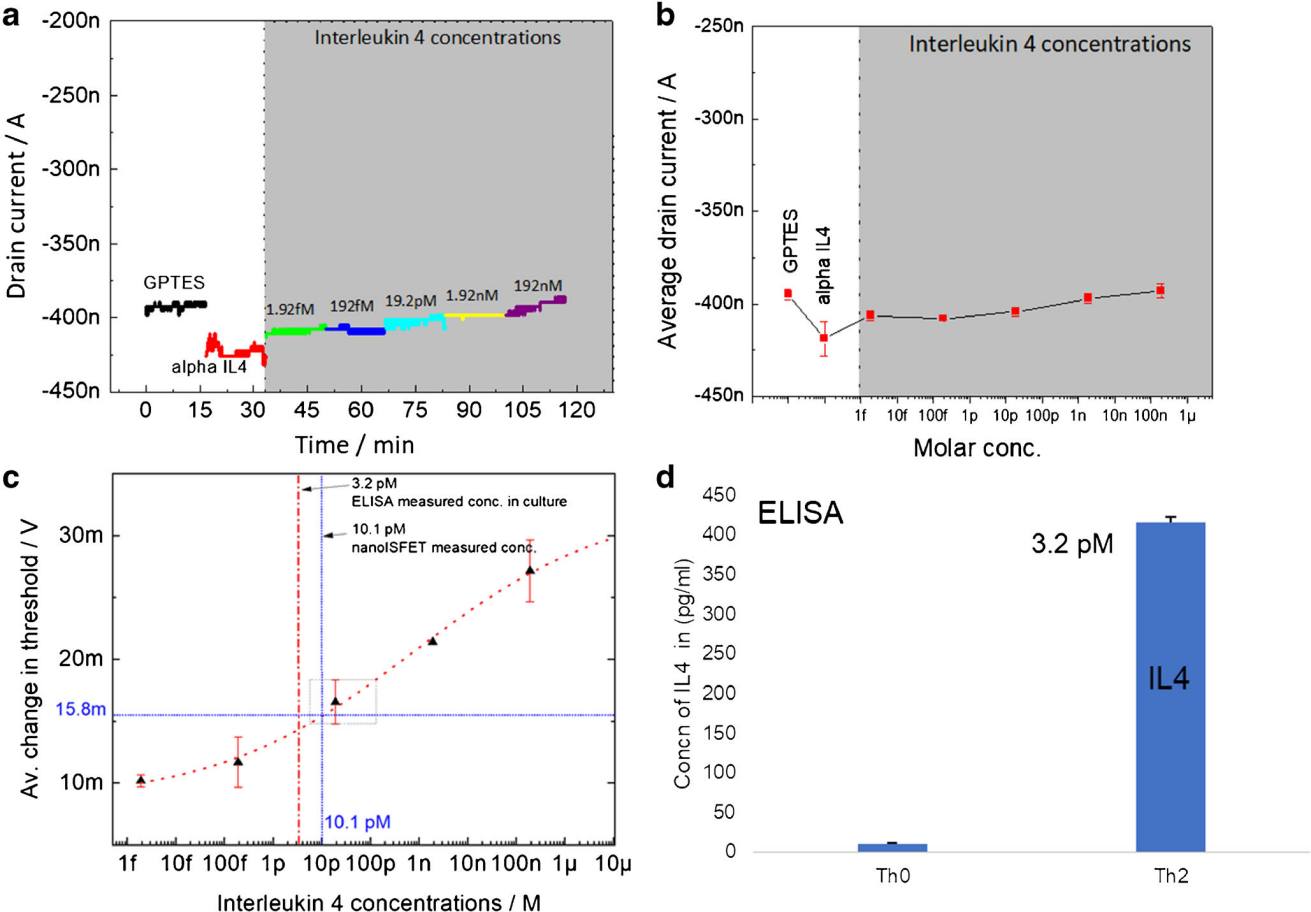
Calibration of the nanoISFETs using known IL-4 concentrations spiked in PBS and real-time detection of IL-4 detection using nanoISFETs. (**a**) The graph shows a typical measurement, where drain output current from a nanoISFET is plotted before and after biofunctionalization steps and then after injections of different concentrations of IL-4 in PBS, (**b**) Average output current for the measurement shown in graph **a**, (**c**) average changes in equivalent threshold values which were calculated from output current values of 4 nanoISFETs for different IL-4 concentrations ranging from 1.92 fM up to 192 nM. The sigmoid fitting of this dose response curve was used for calibration of the sensors to compare the shift in Vth values from mouse T cell culture samples (with “unknown” IL-4 concentration), and (**d**) IL-4 concentrations in an “unknown” sample of mouse T cell culture measured by ELISA

From the measurements shown in Figs. 3 and 4, we conclude that nanoISFETs can be used as a reliable sensor platform for the detection of cytokines in complex culture media. The sensor responses are compared for PoC applications; such nanoISFETs are expected to be an ideal candidate delivering molecular quantification in very short times. In order to evaluate the performance of our nanoISFET immunosensor platform for such applications, we characterized the sensor dynamic ranges and biosensor behavior in real time using our portable sensor readout setup. For this, anti-IL-4-modified nanoISFETs were deployed for time-dependent and quantitative measurements of IL-4. For sensor calibration and in order to avoid expressing precise concentrations of IL-4 in culture media with identical matrix compositions, we decided to use commercially procured IL-4s and prepared different calibration solutions by spiking into PBS. The setup used for the real-time quantitative measurements required no changes from what is shown in Fig. 1c, d. In addition to field-effect measurements, the DC readout tool developed in house is also able to measure an output current at selected working set points as a function of time. IL-4 concentrations of 1.92 fM (equivalent to 2.5 pg/mL), 19.2 fM, 19.2 pM, 1.92 nM, and 192 nM (equivalent to 2.5 μg/mL) in buffer were used for the measurements. The real-time detection of different concentrations of IL-4 is shown in Fig. 5. Figure 5a shows a typical measurement, where the current output from the nanoISFET on *y*-axis is plotted against time while injecting different concentrations of IL-4. All the measurements carried out were performed while applying a fixed Vg to the Ag/AgCl reference electrode at − 1.5 V and a drain source bias of − 1 V. The black curve in the graph represents the drain output of a nanoISFET in PBS for up to 15 min showing a stable electrical response. The rest of the measurements were carried out in continuum, where current output of same biofunctionalized nanoISFET was firstly measured in PBS (red curve) and thereafter stepwise adding different concentrations of IL-4 in PBS. It can be seen that after the biofunctionalization step, the output current decreased significantly, which also reflects the situation shown in Fig. 3, where the field-effect curve shifted towards positive gate voltages. At a constant working point, a shift towards positive gate voltages means a decreased output current. Then after, the current output from the nanoISFET started increasing upon stepwise injecting the IL-4 concentrations. The stepwise increase in output current is also in agreement with the endpoint measurements shown in Fig. 3, where the field-effect curve shifts towards negative gate voltages due to specific binding of IL-4 on the nanoISFET surface. At a constant working point, a shift towards more negative gate voltages would amount to an increased output current. For real-time measurements, each concentration of IL-4 was allowed in the fluidic chamber for 15 to 20 min. Real-time measurements were carried out similarly for other sensor assemblies (see ESM). For determination of sensor behavior in terms of dynamic range and quantification, a dose response curve was worked out after taking an average of current output values for each concentration of IL-4 (Fig. 5b). For this, equivalent Vth values were calculated from average drain outputs by using the equation Vgs = Ids/*g*_m_, (where *g*_m_ represents the transconductance of nanoISFET as calculated from the field-effect characterization of the device at Vgs = − 1.5 V, Vds = − 1 V). Figure 5c shows such equivalent Vth values converted from the output drain current values of the ISFETs against different IL-4 concentrations. The dose response curve is in agreement with the IL-4 measurements carried out in culture media and shows a sigmoidal fitting (red curve) typical of a molecular binding isotherm.

It is to be noted that when measuring IL-4 concentrations in the culture media as shown in Fig. 3, we measured an average change in Vth at − 15.8 mV. This average change in Vth, representative of IL-4 concentrations in Th2 culture, is shown in Fig. 5c by the horizontal dashed line in blue color. The intersection point of this line on the calibration plot relates to IL-4 concentration of 10.1 pM as shown by the vertical bar in blue color. The vertical bar in red with the dash-dot line represents the ELISA reference measurements of IL-4 concentrations in culture media that was used for the measurements as shown in Fig. 3. Figure 5d shows the results from the ELISA measurements used for the determination of IL-4 in Th0 (0.6 pM) and Th2 (3.2 pM) culture media. Therefore, the nanoISFET measurements overestimate the IL-4 concentration as compared with ELISA by about 7 pM, which falls within the error margins of the nanoISFET calibration plot. Even though the electrical measurements come quite close to the results obtained with ELISA, higher accuracy would be needed for practical applications. One cannot directly compare the two methods, for the different surface properties and modification requirements are expected to have an influence. In future, it would be interesting to employ advanced techniques, where ELISA or other optical measurements such as surface plasmon resonance measurements can be carried out simultaneously employing the same surface, i.e., Si/SiO_2_, representing identical biofunctional interfaces and molecular interactions. Here, we assume that the higher ionic strengths and complex compositions of culture media also cause the overestimation of the IL-4 concentrations, since the calibration plot was only worked out in PBS solution without complex biomolecule background.

It is worth mentioning that the development of advanced nanoelectronic biosensor platforms such as presented in this work is mainly driven by the requirements from biomedical research. Active collaborations are fruitful and demanding for us towards the optimization of this novel PoC method and for future protocols towards real biomedical applications. In this respect, we look forward to further develop our nanoISFET platform for molecular screening in clinical settings overcoming concurrent challenges in the field. Use of novel receptors molecules such as aptamers in advanced anti-biofouling matrices on top of sensor surfaces is especially interesting for enhanced charge-based detection for in vivo and in vitro immunosensor applications [7, 55–57]. We have reported earlier on the correlation of interleukin expressions with mRNAs (such as for IL-4 and IL-2 used in this work with the IL-4 and IL-2 for positive and negative controls) in the differentiated T helper cells [48, 54]. Such mRNA correlations are expected to further strengthen on-chip immune strategies such as presented in this work for different pathophysiological conditions and biological development.

## Conclusion

In summary, we present an elegant electrical screening platform that comes with advantages of top-down fabricated nanoISFETs with reproducible sensor behavior and is system-integrated for deployment as PoC biosensors. The nanoISFET-based PoC biosensor platform not only showed an interleukin detection range down to sub-picomolar concentrations in PBS but also demonstrated robust biosensor behavior directly inside complex culture media of mouse T cells. Such miniature electrical detection platforms are expected to enhance bioanalytical screening capabilities in biochemistry and biotechnology domains. In particular, for immunology applications, expression of interleukins panels is associated with complex immune response pathways and as shown in this work, PoC nanoISFETs will be a viable alternative for their capabilities to reduce the cost of screening. Future versions of nanoISFET-based biosensor platforms are expected to take advantage of high-density integration, localized surface modification methods, and developments in readout capabilities, in putting together more compact versions of multiplexed immunosensing devices.

## Electronic supplementary material

ESM 1 (PDF 898 kb)
