# Supplementary material for: Point-of-care-ready nanoscale ISFET arrays for sub-picomolar detection of cytokines in cell cultures
Source: Anal Bioanal Chem. 2020 Jul 28;412(25):6777–88. doi: 10.1007/s00216-020-02820-4 (PMC7496041; doi:10.1007/s00216-020-02820-4)
Supplement: Supplementary file 1 — (PDF 898 kb) [file 216_2020_2820_MOESM1_ESM.pdf]

Analytical and Bioanalytical Chemistry

Electronic Supplementary Material

Point-of-care ready nanoscale ISFET-arrays for sub-picomolar detection of cytokines in cell cultures

Dipti Rani, Yogesh Singh, Madhuri Salker, Xuan Thang Vu, Sven Ingebrandt,
Vivek Pachauri

S1: Isolation of T helper cells and expression of Il4 and IL2

The cytokines IL-4 and IL-2 used in this work were secreted from T helper cells into their culture medium. Naïve T cells were isolated from C57BL/6 mice, which were kept in a conventional pathogen-free facility. Mice aged from 8 to 16 weeks were chosen to isolate naïve T cells ($CD4^{+}$ $CD62L^{high}$ $CD25^{-}$) from their spleen and lymph nodes using the Dynabeads® Untouched™ Mouse CD4 cells kit (from Invitrogen, Thermofisher). First, biotinylated anti-CD25 (7D4; BD Biosciences, Franklin Lakes, NJ) was used to collect the regulatory T cells ($CD4^{+}CD25^{+}$) by positively selection. Finally, biotinylated-anti-CD62L (MEL-14; BD Biosciences) were used for the isolation of naïve T cells. The purity of naïve T-cells was > 90% as determined by flow cytometry measurements (Fig. S1).

Th cell differentiation into Th1, Th2, Th17 and induced Treg (iTreg) types was carried out from naïve T cells as described earlier [1,2]. In brief, naïve T cells ($1.0x10^{6}$) were activated in the presence of plate bound anti-CD3/anti-CD28 antibodies (procured from eBioscience, San Diego, CA) with 1 μg/ml of anti-CD3 and 2 μg/ml of anti-CD28 and were differentiated into Th2 (containing IL-4, IL-5 and IL-10) using 20 ng/ml of recombinant IL-4 (procured from BD Biosciences) and 5 μg/ml of anti-IFN-γ. Control, Th0 differentiation (minimal expression of cytokines of IL-4 and IFN-γ) was carried out using 5 μg/ml of anti-IFN- γ and 5 μg/ml of anti-IL-4 antibodies (both procured from BD Biosciences). T cells were cultured for 3-4 days before staining and analysis of cytokines. After 3-4 days, differentiated Th0 and Th2 cells were treated with 1 μg/ml of phorbol 12-myristate 13-acetate (PMA) and Ionomycin (both from Sigma, Germany) for 4 hours followed with treatment with Brefeldin A (from eBioscience) for 2 hours, cell culture medium was collected and stored at -80 °C for cytokine measurements and PMA, Ionomycin and Brefeldin A treated cells were used for the intracellular staining. Cells were fixed with fixation/permeable buffer (from eBioscience for Foxp3 staining) for 30 minutes before IL-4 intracellular staining.

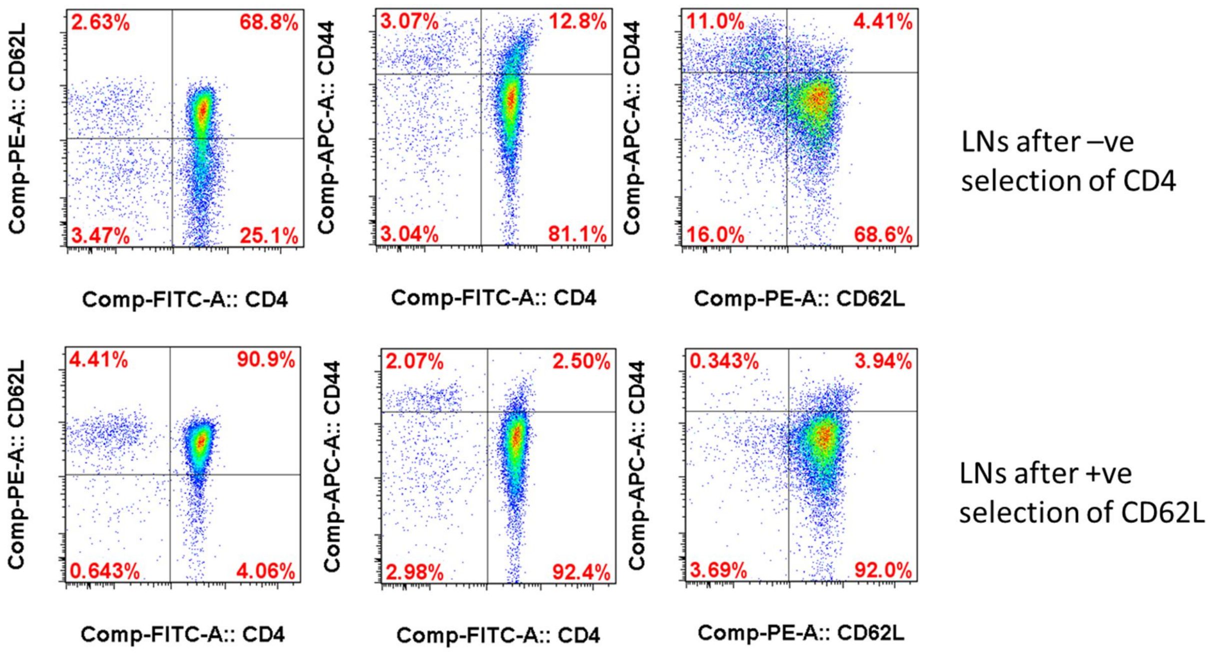

Fig. S1 Lymph nodes (LNs) magnetic purification results shown here for the purity of Naïve $CD4^{+}CD62L^{high}$ cells

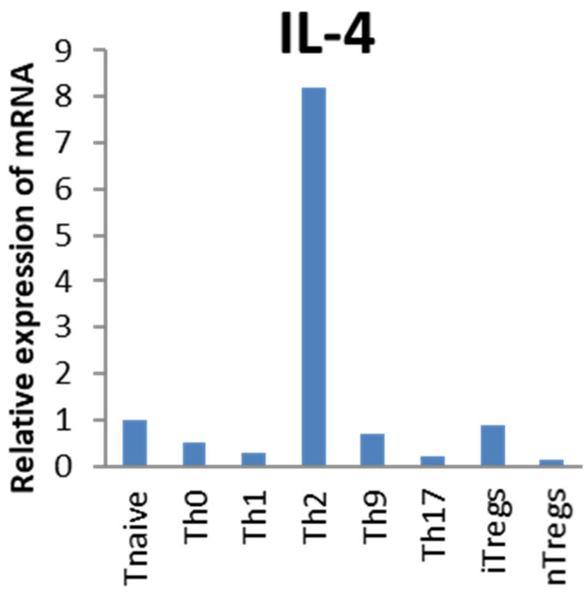

Fig. S2 relative expression of mRNA in T helper cell differentiation cultures are shown in this histogram, where a strong correlation is found for the Th2 cell culture

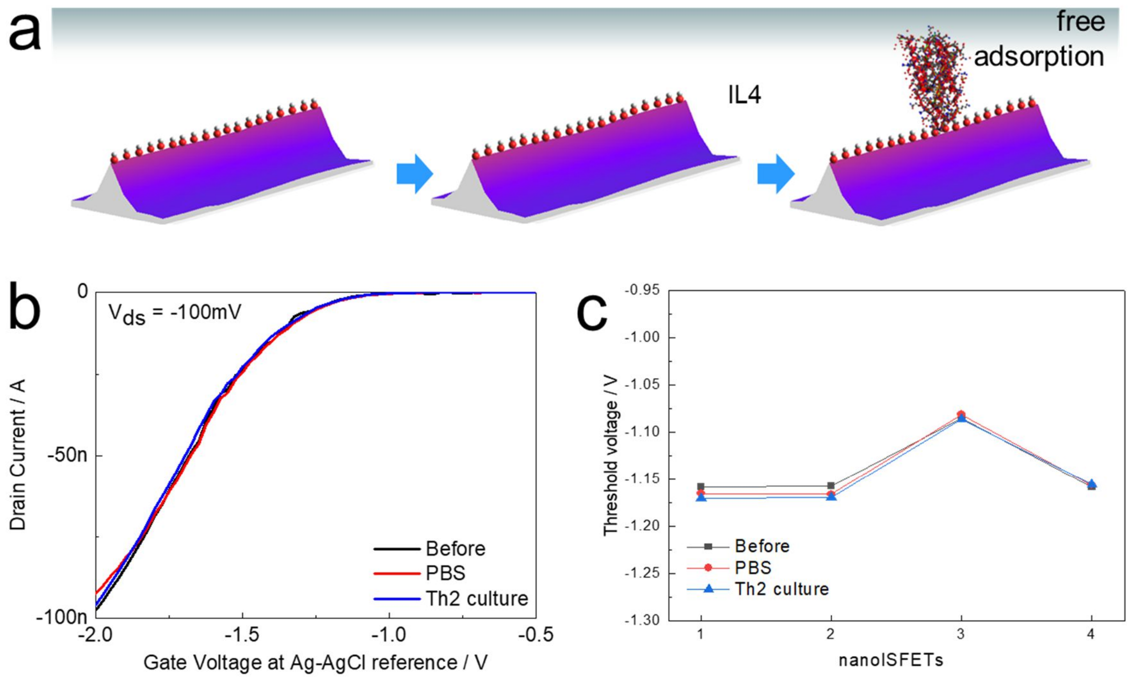

Fig. S3 Field-effect response of the nanoISFET biosensor for non-specific adsorption of IL-4 on the sensor surface. (a) Illustration of the non-specific free adsorption of IL-4 on a nanoISFET, (b,c) field-effect characterizations in PBS (with no IL4) and Th2 cell cultures (with IL-4) did not show any significant changes in Vth

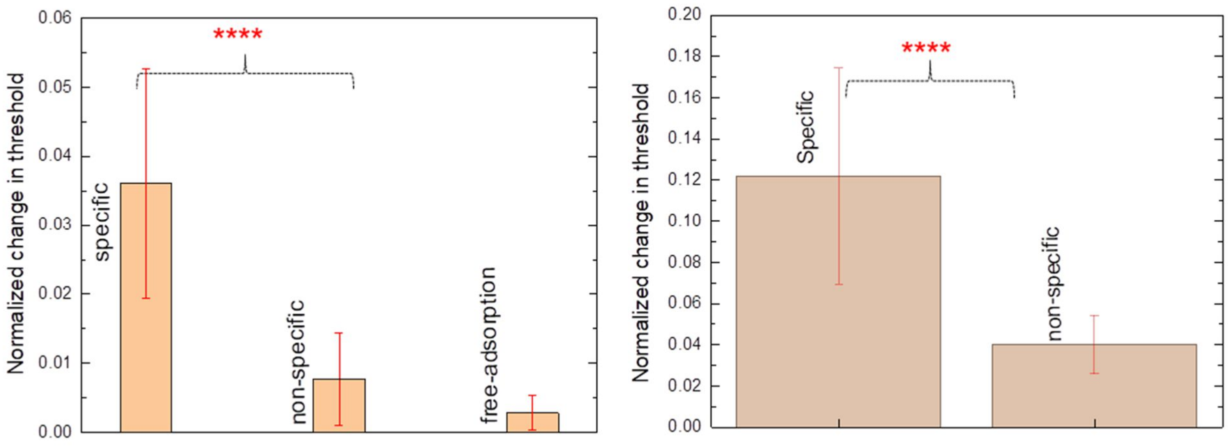

Fig. S4 Left side graph showing normalized changes in the Vth values of anti-IL-4 modified nanoISFETs for specific binding (culture with IL-4), non-specific binding (culture with other cytokines but negligible concentrations of IL-4) and free-adsorption of IL-4s on non-modified nanoISFETs. An unpaired T-test analysis of mean values for specific and non-specific binding showed the sensor signal as statistically extremely significant with P values of 0.0001. The right side graph showing normalized changes in Vth of anti-IL-2 modified nanoISFETs for specific binding (culture with IL-2), non-specific binding (culture with other cytokines, but negligible concentrations of IL-2). An unpaired T-test analysis of mean values for specific and non-specific binding showed the sensor signal as statistically extremely significant with P values of 0.0001

Normalized changes in the V_{th} were extracted by -

$$\frac{\Delta V_{th}}{V_{th}} = \frac{V_{th}\,(Th2) - V_{th}\,(anti - IL - 4)}{V_{th}(anti - IL - 4)}$$

Similarly, normalized changes in V_{th} were evaluated for IL-2 (Itregs) immobilization and other control experiments. We found significant difference in the normalized ΔV_{th} values for IL4 and other two control experiments (non-specific & free-adsorption), which indicated specific response of the developed nanoISFET platform. We have changed the histogram plots in figure 3d, and 4d (shown above) to illustrate the normalized changes in threshold voltages.

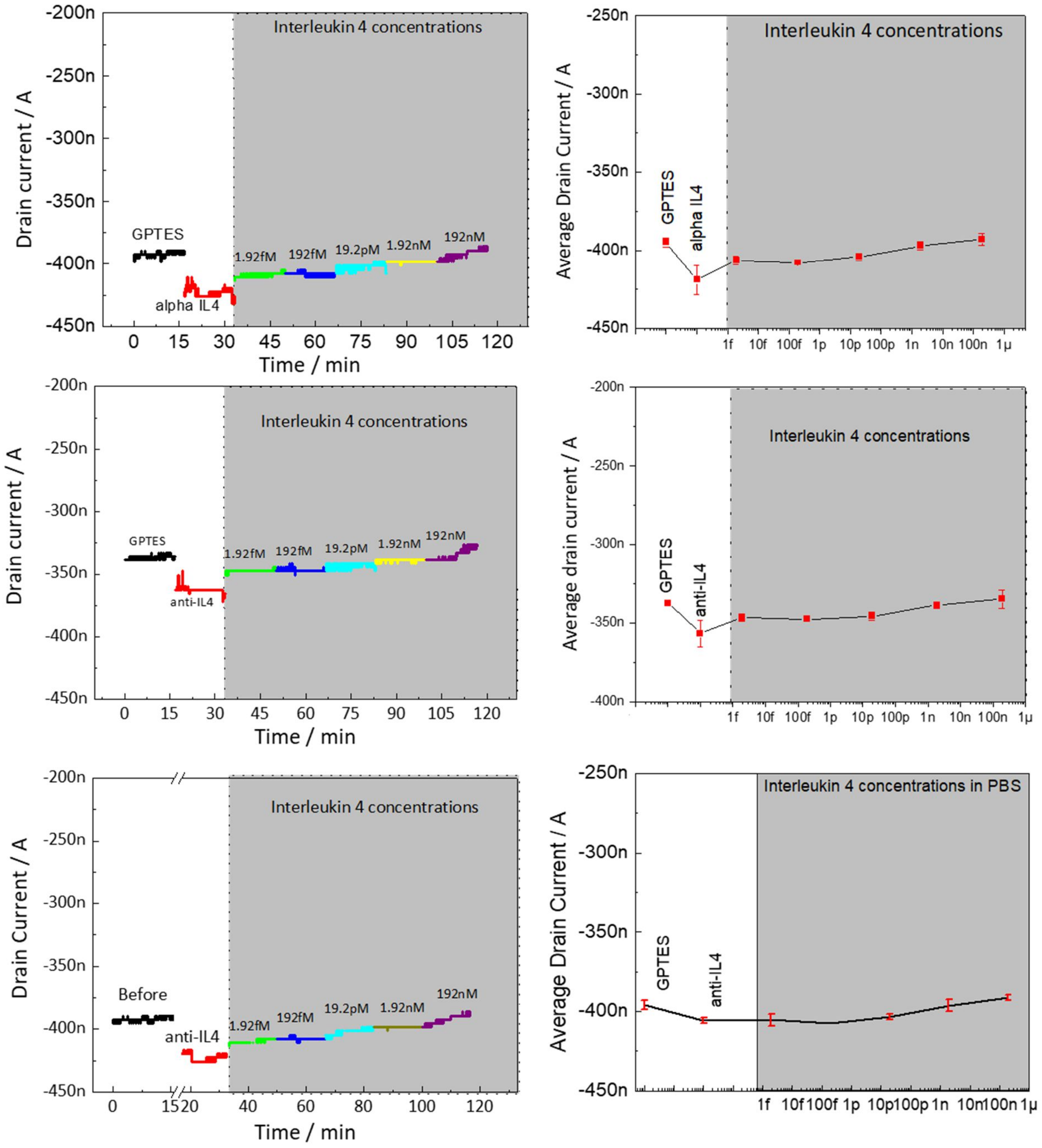

Fig. S5 Typical real-time measurements for detection of IL-4 are shown here with three different nanoISFETs.

References

[1] Singh Y, Garden OA, Lang F, Cobb BS. Retroviral Transduction of Helper T Cells as a Genetic Approach to Study Mechanisms Controlling their Differentiation and Function. J. Vis. Exp. 2016;117:e54698.

[2] Singh Y, Garden OA, Lang F, Cobb BS. MicroRNA-15b/16 Enhances the Induction of Regulatory T Cells by Regulating the Expression of Rictor and mTOR. J Immunol. 2015;195:5667-5677.
